# Suppression of *FoxO1* mRNA by β_2_‐adrenoceptor–cAMP signaling through miR‐374b‐5p and miR‐7a‐1‐3p in C2C12 myotubes

**DOI:** 10.1002/2211-5463.13368

**Published:** 2022-02-07

**Authors:** Saki Shimamoto, Kazuki Nakashima, Nao Nishikoba, Rukana Kohrogi, Akira Ohtsuka, Shinobu Fujimura, Daichi Ijiri

**Affiliations:** ^1^ Department of Agricultural Sciences and Natural Resources Kagoshima University Japan; ^2^ The United Graduate School of Agricultural Sciences Kagoshima University Japan; ^3^ 12978 Graduate School of Science and Technology Niigata University Japan; ^4^ Division of Meat Animal and Poultry Research Institute of Livestock and Grassland Science NARO Tsukuba Japan

**Keywords:** FoxO, microRNA, skeletal muscle, ubiquitin ligase, β_2_‐adrenoceptor

## Abstract

β_2_‐Adrenoceptor (β_2_‐AR) signaling decreases the transcriptional activity of forkhead box O (FoxO), but the underlying mechanisms remain incompletely understood. Here, we investigated how β_2_‐AR signaling regulates the protein abundance of FoxO and its transcriptional activity in skeletal muscle. We observed that stimulation of β_2_‐AR with its selective agonist, clenbuterol, rapidly decreased *FoxO1* mRNA expression, and this was accompanied by a decrease in either FoxO1 protein level or FoxO transcriptional activity. We subsequently observed that miR‐374b‐5p and miR‐7a‐1‐3p were rapidly upregulated in response to β_2_‐AR stimulation. Transfection with mimics of either miRNA successfully decreased *FoxO1* mRNA. Moreover, because β_2_‐AR stimulation increased cAMP concentration, pretreatment with an adenylyl cyclase inhibitor canceled out these effects of β_2_‐AR stimulation. These results suggest that β_2_‐AR stimulation results in rapid upregulation of miR‐374b‐5p and miR‐7a‐1‐3p in myotubes, which in turn results in a decrease in *FoxO1* mRNA expression via the β_2_‐AR–cAMP signaling pathway.

## Graphical abstract

We investigated how β_2_‐adrenoceptor (β_2_‐AR) signaling regulates the protein abundance of forkhead box O (FoxO) and its transcriptional activity in skeletal muscle. β_2_‐AR stimulation resulted in rapid upregulation of miR‐374b‐5p and miR‐7a‐1‐3p in myotubes and consequently decreased *FoxO1* mRNA expression via the β_2_‐AR–cAMP signaling pathway.

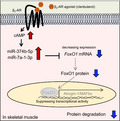

**Abbreviations**
3′UTR: 3′ untranslated region
cAMP: cyclic adenosine monophosphate
DMEM: Dulbecco’s modified Eagle’s medium
FoxO: forkhead box O
GAPDH: glyceraldehyde 3‐phosphate dehydrogenase
HuR: RNA‐binding protein Elav/Hu
miRNA: microRNA
P/S: penicillin/streptomycin
PBS: phosphate‐buffered saline
PKA: protein kinase A
QKI: RNA‐binding protein Quaking
SE: standard error
Ub: ubiquitin
β_2_‐AR: β_2_‐adrenoceptor

Skeletal muscle mass is controlled through a delicate balance between protein synthesis and protein degradation [1]. Changes in the rate of protein degradation may contribute to either normal muscle growth or muscle atrophy [2, 3]. In addition, the rate of protein degradation is probably regulated through the ubiquitin (Ub)–proteasome system [4, 5, 6]. In this system, proteins destined for degradation are covalently linked to a chain of Ub molecules, which marks them for breakdown by the 26S proteasome [7, 8]. Because the mRNA expression of Ub ligases clearly correlates with polyubiquitination, they play an important role in controlling polyubiquitination, a rate‐limiting step in the Ub–proteasome system [7, 9, 10].

The forkhead box O (FoxO) transcription factors have a critical role in the transcriptional regulation of Atrogin‐1/MAFbx, one of the muscle‐specific Ub ligases, and thus, they play an important role in controlling the degradation of muscle protein [11]. The transcriptional activity of FoxO proteins is also affected by a variety of post‐translational modifications (e.g., phosphorylation, acetylation, and mono‐ and polyubiquitination) [12, 13]. Phosphorylation of FoxO protein changes its subcellular localization from the nucleus to the cytosol, decreases its DNA binding activity, and thereby suppresses its transcriptional activity [13, 14]. It is well known that the insulin and insulin‐like growth factor‐I/AKT pathway suppresses FoxO transcriptional activity and expression of the mRNAs for *Atrogin‐1/MAFbx* by AKT‐mediated phosphorylation [15, 16, 17, 18, 19].

In skeletal muscle, either epinephrine or norepinephrine decreases the rate of Ub–proteasome system‐dependent protein degradation by reducing the expression of *Atrogin‐1/MAFbx* mRNA [20, 21]. Three β‐adrenergic receptor (β‐AR) subtypes have been identified, β_1_‐AR, β_2_‐AR, and β_3_‐AR, and the injection of β_2_‐AR agonists (e.g., clenbuterol, terbutaline, and formoterol) also reduces the gene expression of Atrogin‐1/MAFbx, consequently exerting an anabolic effect [22, 23]. Our previous study suggested that a β_2_‐AR selective agonist, clenbuterol, suppressed the transcriptional activity of FoxO by AKT‐related phosphorylation [24]. Recently, the injection of norepinephrine was shown to suppress FoxO transcriptional activity via the cAMP–protein kinase A (PKA) pathway, consequently decreasing the expression of *Atrogin‐1/MAFbx* mRNA in murine skeletal muscles [23]. Interestingly, Silveria et al. [25] also reported that norepinephrine stimulation induces FoxO1 phosphorylation and decreases FoxO1 protein abundance. The abundance of FoxO protein also has dramatic effects on its own transcriptional activity and/or the functions of its downstream targets [26, 27]. These findings raised the possibility that either β_2_‐AR agonists or norepinephrine regulates FoxO1 transcriptional activity by controlling not only phosphorylation level but also protein abundance in skeletal muscle. However, the mechanisms behind the adrenergic signaling‐induced downregulation of FoxO protein abundance in skeletal muscle remain unclear.

On the basis of these findings, to elucidate the role of FoxO in controlling β_2_‐AR‐induced suppression of muscle protein degradation, this study investigated how β_2_‐AR signaling regulates the protein abundance of FoxO and its transcriptional activity in skeletal muscle.

## Materials and methods

### Cell cultures

Murine myoblasts (C2C12, ATCC^®^ CRL‐1772™) were purchased from ATCC (Manassas, VA, USA) and cultured in Dulbecco’s modified Eagle’s medium (DMEM) supplemented with 10% fetal bovine serum and 1% penicillin/streptomycin (P/S) for 3 days. Cells were grown in 10 cm dish (for measurement of N^τ^‐methylhistidine release and protein content) or 6‐well plate (for all other experiments) at 37 °C in 5% (v/v) CO_2_ in a humidified environment. To induce myotube formation, the medium was replaced with DMEM supplemented with 2% horse serum and 1% P/S for 5 days. All drugs were made up in phosphate‐buffered saline (PBS) and subsequently added to the appropriate tissue culture wells (1 : 1000). The vehicle‐control wells received just PBS in the same volume as the treatment groups. To study the effects of β_2_‐AR agonist treatment, C2C12 myotubes were treated with clenbuterol (1 μm), because this concentration of clenbuterol has been reported to successfully decrease *Atrogin‐1/MAFbx* mRNA expression in C2C12 myotubes [28]. In addition, an inhibitor of adenylyl cyclase (SQ22536, 10 μm) was used to block cAMP synthesis.

### RNA extraction and quantitative real‐time polymerase chain reaction (PCR)

C2C12 myotubes were homogenized in ISOGEN II (Nippon Gene, Tokyo, Japan), in accordance with the manufacturer’s instructions. Real‐time PCR was performed as described previously [24]. Each sample was run in duplicate with no template or negative RT controls in each plate. Efficiencies and *R* ^2^ were assessed using five‐point cDNA serial dilution: PCRs were highly specific and reproducible (0.96 < *R* ^2^ < 1.05) and all primer pairs had equivalent PCR efficiency (from 98% to 105%). The melting curves revealed a single peak for all primer pairs. The coefficient of variation was 7–11%. Amplification, dissociation curves, and gene expression analysis were performed using the Dissociation Curves software (Applied Biosystems, Foster City, CA, USA). The level of *18S ribosomal RNA* was used as an internal standard. The primers used in this study are listed in Table 1. Each gene expression result is expressed as the ratio of the experimental treatment value to the corresponding untreated control value.

**Table 1:** List of primer sequence used for quantitative real‐time polymerase chain reaction.

Gene	Sequence (5'‐3')
*FoxO1*	
Forward	GCT GGG TGT CAG GCT AAG AG
Reverse	GGA CTG CTC CTC AGT TCC TG
*Atrogin‐1/MAFbx*	
Forward	GCA AAC ACT GCC ACA TTC TCT C
Reverse	CTT GAG GGG AAA GTG AGA C
*18s ribosomal RNA*	
Forward	ACT CAA CAC GGG AAA CCT CAC C
Reverse	CCA GAC AAA TCG CTC CAC CAA C

### Antibodies

Anti‐FoxO1 (#9454) and anti‐phospho‐FoxO1 (Ser256, #9461) were purchased from Cell Signaling Technology (Beverly, MA, USA). Anti‐glyceraldehyde 3‐phosphate dehydrogenase (GAPDH, sc‐20357), anti‐rabbit IgG (sc‐2030), and anti‐goat IgG (sc‐2020) were purchased from Santa Cruz Biotechnology (Santa Cruz, CA, USA).

### Protein extraction and western blot analysis

C2C12 myotubes were homogenized in 500 μL of a lysis buffer consisting of 50 mm Tris, 150 mm NaCl, 0.5% sodium deoxycholate, 0.1% sodium dodecyl sulfate, 1 mm ethylenediaminetetraacetic acid, and 1% protease inhibitor cocktail (25955‐11; Nacalai Tesque, Kyoto, Japan), pH 8.0. The lysate was centrifuged at 20 000 ***g*** for 10 min at 4 °C, and the supernatant was collected.

Western blot analysis was performed as described previously [24]. In brief, samples were electrophoresed on an SDS/10% (w/v) polyacrylamide gel and then transferred to a polyvinylidene difluoride membrane (IPVH00010; Millipore Co., Billerica, MA, USA). The membrane was blocked with Bullet Blocking One (13779‐01; Nacalai Tesque) for 10 min at room temperature. Subsequently, the blocked membrane was incubated with primary antibody in Can Get Signal I (Toyobo, Osaka, Japan) overnight at 4 °C (1 : 5000 dilution). Then, these membranes were incubated with a secondary antibody in Can Get Signal II at 37 °C for 2 h (1 : 5000 dilution). The blots were detected using Western Blotting Detection Reagent (2332637; ATTO Co., Tokyo, Japan), in accordance with the manufacturer’s instructions. Next, all membranes were incubated with primary antibody against GAPDH in Can Get Signal I overnight at 4 °C (1 : 5000 dilution). Then, these membranes were incubated with a secondary antibody against goat IgG in Can Get Signal II at 37 °C for 2 h (1 : 5000 dilution). The blots were detected using the Western Blotting Detection Reagent. Relative band intensity was quantified using imagej software (National Institutes of Health, Bethesda, MD, USA).

### Luciferase assay

C2C12 myotubes were transfected with the FoxO reporter and negative control reporter (60643; BPS Bioscience, San Diego, CA, USA) using the TransIT‐X2 Dynamic Delivery System. Fold induction of normalized reporter activity by FoxO (ratio of normalized reporter activity in the presence of clenbuterol to that in the presence of the negative control expression vector) in C2C12 myotube culture medium after 3 h of clenbuterol stimulation was calculated. The myotubes were also transfected with miRNA mimic (negative control, miR‐374b‐5p, or miR‐7a‐1‐3p). At 24 h post‐transfection, luciferase activity was measured with the Dual‐Glo^®^ Luciferase Assay System (Promega, Madison, WI, USA) and normalized to the internal control.

### Measurement of N^τ^‐methylhistidine release in the medium of C2C12 myotubes

C2C12 myotubes were incubated with clenbuterol (1 μm) for 24 h in DMEM. The medium was then collected, and N^τ^‐methylhistidine concentration was determined as described previously [29]. Culture medium was mixed with 20% sulfosalicylic acid and centrifuged at 10 000 ***g*** for 5 min. The supernatant was recovered and evaporated under reduced pressure. The residue was dissolved in 0.2 m pyridine and applied to a cation‐exchange column (7 × 60 mm, Dowex 50W‐X8, 200–400 mesh, pyridine form). After most of the acidic and neutral amino acid had been washed out with 0.2 m pyridine, N^τ^‐methylhistidine was eluted with 1 m pyridine and collected. The solvent was evaporated, and the residue was dissolved in mobile phase (15 mm sodium 1‐octanesulfonate in 20 mm KH_2_PO_4_). An aliquot was injected into an HPLC system (LC‐2000 Plus HPLC System; JASCO Co. Ltd., Tokyo, Japan) equipped with an Inertsil ODS‐80A column (4.6 × 250 mm, 5 μm; GL Sciences, Tokyo, Japan). The column was attached to an oven at 50 °C. A fluorometric detector set at an excitation wavelength of 365 nm and an emission wavelength of 460 nm was used to monitor the fluorescence derived from the reaction with ortho‐phthalaldehyde. The protein content was determined by the Bradford method with bovine serum albumin as a standard [30].

### Measurement of protein content of C2C12 myotubes

C2C12 myotubes were incubated with clenbuterol (1 μm) for 24 h in DMEM. Total protein and DNA were simultaneously isolated from myotubes using ISOGEN™ reagent (Nippon Gene), in accordance with the manufacturer’s instructions. The protein content was determined by the Bradford method with bovine serum albumin as a standard [30] and normalized by DNA content, which was determined using the Nanodrop Lite spectrophotometer (Thermo Fisher Scientific, Waltham, MA, USA) readings at 260 nm.

### miRNA extraction, microarray analysis, and miRNA expression

Using the mirVana™ miRNA Isolation Kit (Ambion, Austin, TX, USA), miRNAs were isolated in accordance with the manufacturer’s instructions. All samples were diluted to a final concentration of 3 ng·μL^−1^. The samples were used immediately or stored at −80 °C until use, and miRNA microarray assays were outsourced to Filgen Inc. (Nagoya, Japan). Microarray analysis of miRNAs was performed using the Affymetrix GeneChip^®^ miRNA 4.0 Array (Thermo Fisher Scientific). These arrays were scanned with the GeneChip^®^ Scanner 3000 7G (Thermo Fisher Scientific). Differentially expressed miRNAs were analyzed using the Affymetrix^®^ Expression Console™ Software (Thermo Fisher Scientific).

The miRNAs were reverse transcribed using the Taqman^®^ miRNA reverse transcription kit (Applied Biosystems), and real‐time PCR of miRNAs was performed using the TaqMan Universal Master Mix II (Applied Biosystems), in accordance with the manufacturer’s instructions. Quantitative analysis of the expression was carried out using the TaqMan Assay Kit (Applied Biosystems). The level of snoRNA142 was used as an internal standard. The Taqman assay IDs used in this study are as follows: snoRNA142 (Taqman assay ID: 001231), miR‐7a‐1‐3p (Taqman assay ID: 001338), and miR‐374b‐5p (Taqman assay ID: 001319). Each miRNA expression result is expressed as the ratio of the experimental treatment value to the corresponding untreated control value.

### Transfection with small interfering RNAs

Transient transfection of C2C12 cells was performed using the TransIT‐X2 Dynamic Delivery System (Mirus, Madison, WI, USA), in accordance with the manufacturer’s instructions. Briefly, 30 μm mirVana miRNA mimic (miR‐374b‐5p, miR‐7038‐3p, miR‐7016‐3p, or miR‐7a‐1‐3p) or AccuTarget™ miRNA Mimic Negative Control #1 (4464058, Ambion) was separately used in each transfection. The assay IDs of mirVana miRNA mimics used in this study are as follows: miR‐374b‐5p (MC11339), miR‐7038‐3p (MC26508), miR‐7016‐3p (MC30144), and miR‐7a‐1‐3p (MC10440). After transfection, cells were added to prewarmed medium and immediately placed in an incubator at 37 °C and 5% CO_2_. Cells were harvested at 24 h after transfection.

### cAMP levels in C2C12 myotubes

cAMP levels were determined using an Enzyme Immuno Assay Kit (Cayman Chemical, Ann Arbor, MI, USA), in accordance with the manufacturer’s instructions.

### Statistical analysis

The data are expressed as mean ± standard error (SE). Statistical comparisons were performed using Student’s *t*‐test, Dunnett’s test, or Tukey’s multiple comparison test. *P* values under 0.05 or 0.01 were considered to indicate statistical significance. These analyses were performed using r software (version 4.1.1, Free Software, https://www.r‐project.org).

## Results

### β_2_‐AR agonist, clenbuterol, reduces protein degradation followed by suppression of *FoxO1* mRNA expression in myotubes

**Fig. 1:**
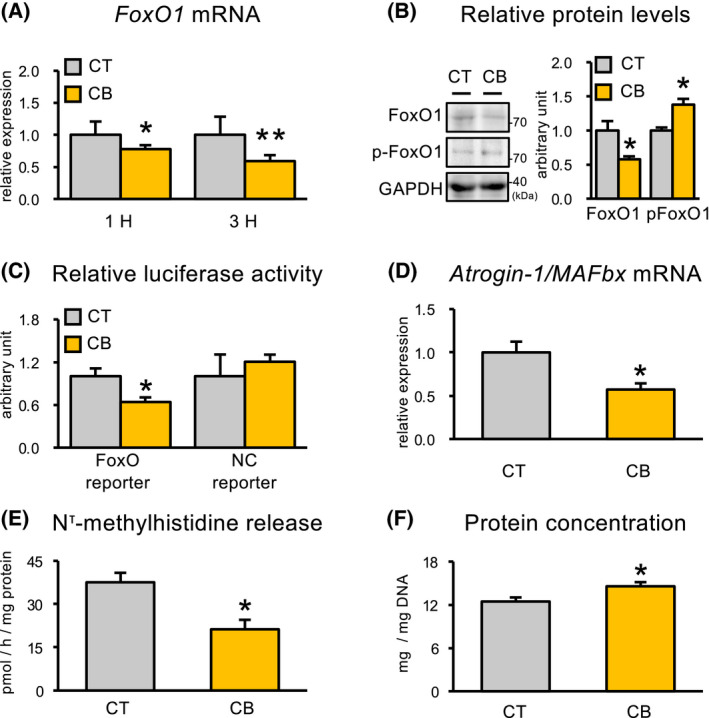
β_2_‐AR stimulation reduces *FoxO1* mRNA expression accompanied by decreasing FoxO transcriptional activity in C2C12 myotubes. Quantitative real‐time PCR analysis of *FoxO1* mRNA after 1 and 3 h of clenbuterol stimulation (A). Protein levels of total FoxO1 and Phospho‐Foxo1 in C2C12 myotube culture medium after 3 h of clenbuterol stimulation (B). Fold induction of normalized reporter activity by FoxO (ratio of normalized reporter activity in the presence of clenbuterol to that in the presence of the negative control expression vector) in C2C12 myotube culture medium after 3 h of clenbuterol stimulation (C). Quantitative real‐time PCR analysis of *Atrogin‐1/MAFbx* mRNA (D). The rates of N^τ^‐methylhistidine release from C2C12 myotubes (E) and protein concentration of C2C12 myotubes (F) after 24 h of clenbuterol stimulation. *P < 0.05 (versus control). **P < 0.01 (versus control), Student's *t*‐test. Values are mean ± standard error (*n* = 6). Atrogin‐1/MAFbx, muscle atrophy F‐box; CB, clenbuterol; CT, control; FoxO, forkhead box O; GAPDH, glyceraldehyde 3‐phosphate dehydrogenase; β_2_‐AR, β_2_‐adrenoceptor.

β_2_‐AR activation by 1 μm clenbuterol resulted in decreases of *FoxO1* mRNA expression after 1 and 3 h of stimulation (Fig. 1A). In agreement with the decreased mRNA expression, decrease in FoxO1 protein levels was confirmed 3 h after clenbuterol stimulation, while phosphorylated FoxO1 protein levels were increased (Fig. 1B). The transcriptional activity of FoxO protein was determined by a luciferase reporter assay, in which the gene for luciferase was under the control of the FoxO‐response element. FoxO‐specific reporter assays showed that β_2_‐AR activation by 1 μm clenbuterol resulted in decreased FoxO transcriptional activity after 3 h (Fig. 1C). In addition, clenbuterol stimulation significantly decreased the mRNA expression of *Atrogin‐1/MAFbx*, which is a transcriptional target of FoxO1, after 3 h of stimulation (Fig. 1D). Furthermore, the release of N^τ^‐methylhistidine, as an index of muscle protein degradation, was decreased in C2C12 myotubes 24 h after clenbuterol stimulation, while the protein concentration of C2C12 myotubes 24 h after clenbuterol stimulation was higher than that of the control myotubes (Fig. 1E,F).

### Profiling miRNA expression changes in response to β_2_‐AR stimulation

**Fig. 2:**
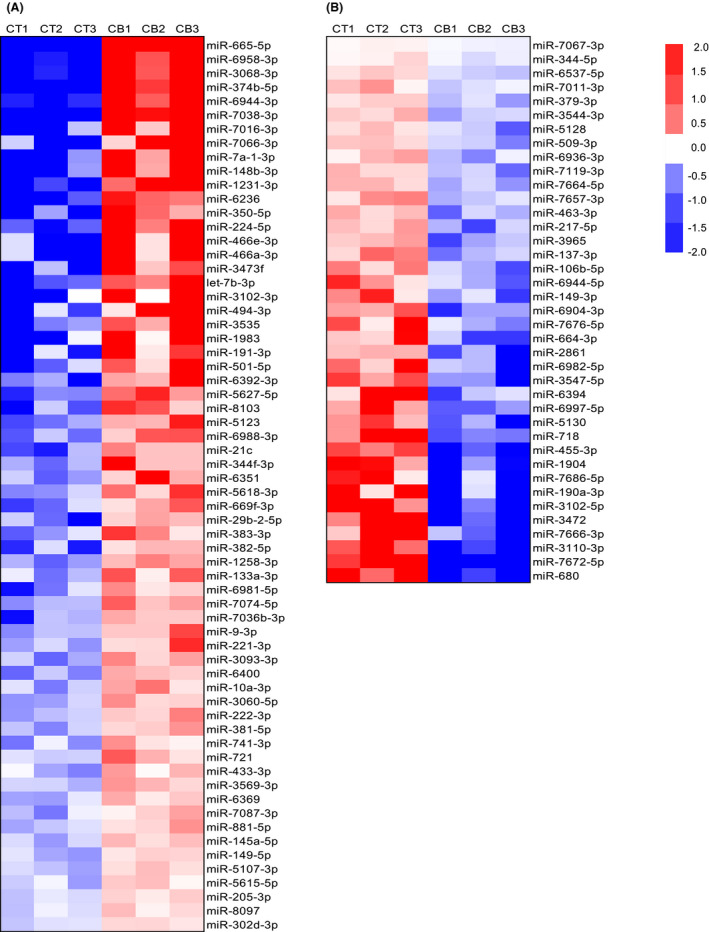
Microarray analysis of miRNA expression in C2C12 myotubes with or without β_2_‐AR stimulation. Overall, 64 miRNAs were upregulated (A) and 39 were downregulated in clenbuterol‐stimulated myotubes (B). Blue and red denote lower and higher expression, respectively. miRNAs whose levels changed significantly (*P* < 0.05) after 1 h of stimulation with the β_2_‐AR agonist clenbuterol are shown (*n* = 3). CB, clenbuterol; CT, control; β_2_‐AR, β_2_‐adrenoceptor.

We performed a microarray analysis to identify miRNAs that were differentially expressed between C2C12 myotubes stimulated with 1 μm clenbuterol for 1 h and their control myotubes (GSE130181). We found that 3142 miRNAs were detectable in these myotubes. The differentially expressed miRNAs among them are shown as heat maps in Fig. 2. In total, 103 miRNAs were differentially expressed; 64 miRNAs were upregulated in clenbuterol‐stimulated myotubes (Fig. 2A), and 39 were downregulated (Fig. 2B).

### Identification and validation of miRNA interaction sites in the 3′ untranslated region (3′UTR) of *FoxO1*

**Fig. 3:**
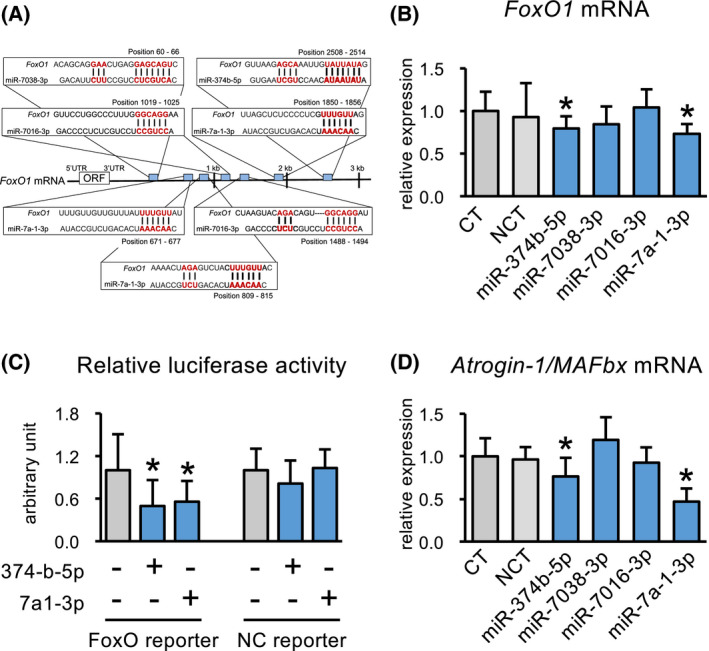
miRNA target sites in the 3′UTR of *FoxO1* and effects of miRNA mimics on *FoxO1* and *Atrogin‐1/MAFbx* expression. Predicted miR‐374b‐5p, miR‐7038‐3p, miR‐7016‐3p, and miR‐7a‐1‐3p target sites in the 3′UTR of the *FoxO1* transcript (A). Quantitative real‐time PCR analysis of *FoxO1* mRNA expression in C2C12 myotubes transfected with miR‐374b‐5p, miR‐7038‐3p, miR‐7016‐3p, or miR‐7a‐1‐3p mimics or the NCT negative control (B). Fold induction of normalized reporter activity by FoxO (ratio of normalized reporter activity in the presence of miR‐374b‐5p or miR‐7a‐1‐3p mimics to that in the presence of the negative control expression vector) (C). Quantitative real‐time PCR analysis of *Atrogin‐1/MAFbx* mRNA expression (D). Values are expressed relative to the mean value for the control group. *P < 0.05 (versus control), Dunnett's test. Values are mean ± standard error (*n* = 6). Atrogin‐1/MAFbx, muscle atrophy F‐box; CT, untransfected control; FoxO, forkhead box O; NCT, negative control small interfering RNA.

We focused on nine miRNAs that were upregulated more than 2.5‐fold in clenbuterol‐stimulated C2C12 myotubes compared with the levels in controls. We used the prediction algorithm TargetScan to identify miRNAs having candidate binding sites within the 3′UTR of *FoxO1*. As shown in Fig. 3A, this analysis predicted that the 3′UTR of *FoxO1* might be targeted by four of the nine miRNAs (each having one to three putative binding sites). We therefore focused further on these four miRNAs: miR‐374b‐5p, miR‐7038‐3p, miR‐7016‐3p, and miR‐7a‐1‐3p. To investigate their functional roles, we examined *FoxO1* expression in C2C12 myotubes transfected with their mimics. Among them, transfection with the miR‐374b‐5p and miR‐7a‐1‐3p mimics successfully decreased *FoxO1* mRNA expression in the C2C12 myotubes (Fig. 3B). Concomitantly, the miR‐374b‐5p and miR‐7a‐1‐3p mimics also decreased the transcriptional activity of FoxO protein in response to miR‐374b‐5p and miR‐7a‐1‐3p, as determined by a luciferase reporter assay. In the C2C12 myotubes, transfection with the miR‐374b‐5p and miR‐7a‐1‐3p mimics resulted in a significant decrease in the relative luciferase activity compared with that of their control myotubes 24 h after transfection (Fig. 3C). In concert with FoxO1 protein levels, transfection with either miR‐374b‐5p or miR‐7a‐1‐3p mimic decreased *Atrogin‐1/MAFbx* mRNA expression (Fig. 3D).

### The inhibition of cAMP synthesis canceled out β_2_‐AR agonist‐induced decrease in *FoxO1* mRNA expression

**Fig. 4:**
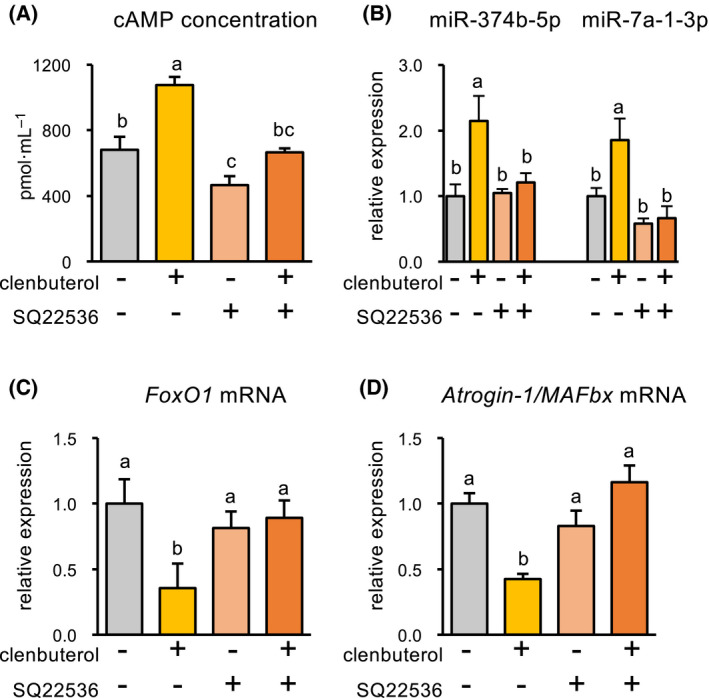
The inhibition of cAMP synthesis cancels out β_2_‐AR agonist‐induced decrease in *FoxO1* mRNA expression. cAMP concentrations in C2C12 myotubes treated with clenbuterol and/or an adenylyl cyclase inhibitor (SQ22536) (A). Quantitative real‐time PCR analysis of miR‐374b‐5p and miR‐7a‐1‐3p (B), *FoxO1* mRNA (C), *Atrogin‐1/MAFbx* mRNA (D). Means with different letters are significantly different (*P* < 0.05), Tukey’s multiple comparison test. Values are expressed as mean ± SE (*n* = 6). Atrogin‐1/MAFbx, muscle atrophy F‐box; cAMP, cyclic adenosine monophosphate; FoxO, forkhead box O; GAPDH, glyceraldehyde 3‐phosphate dehydrogenase; β_2_‐AR, β_2_‐adrenoceptor.

Clenbuterol stimulation significantly increased the cAMP concentration in C2C12 myotubes after 1 h of stimulation, while this effect was not observed in C2C12 myotubes pretreated with an adenylyl cyclase inhibitor (SQ22536) before clenbuterol stimulation (Fig. 4A). In addition, although clenbuterol stimulation increased the expression levels of *miR‐375b‐5p* and *miR‐7a‐1‐3p*, neither of these was observed in C2C12 myotubes pretreated with SQ22536 before clenbuterol stimulation (Fig. 4B).

Clenbuterol stimulation significantly decreased the expression level of *FoxO1* mRNA, while this was not observed in C2C12 myotubes pretreated with SQ22536 (Fig. 4C). In concert with this, *Atrogin‐1/MAFbx* mRNA expression was decreased by clenbuterol stimulation, while this was not observed in C2C12 myotubes pretreated with SQ22536 (Fig. 4D).

## Discussion

The data obtained in this study demonstrate that β_2_‐AR stimulation acutely suppressed *FoxO1* mRNA expression and consequently reduced FoxO1 protein abundance in C2C12 myotubes. In addition, β_2_‐AR stimulation also increased the phosphorylation of FoxO1 proteins in the myotubes. These results suggest that β_2_‐AR stimulation suppressed FoxO1 transcriptional activity via the dual mechanisms of altering FoxO1 protein abundance and phosphorylation in skeletal muscles and consequently suppresses muscle protein degradation by decreasing *Atrogin‐1/MAFbx* mRNA expression in skeletal muscle.

It has been reported that the half‐life of *FoxO1* mRNA was calculated to be 4.9 h in murine cardiomyocytes [31]; however, β_2_‐AR stimulation decreased *FoxO1* mRNA 1 h after stimulation in C2C12 myotubes in this study. This raised the possibility that β_2_‐AR activation might destabilize *FoxO1* mRNA in C2C12 myotubes. Numerous miRNAs are known to influence the evolution and stability of many mRNAs and to adjust protein output [32]. During the last decade, many miRNAs have been identified as post‐transcriptional regulators of FoxO synthesis [33]. In this study, β_2_‐AR activation acutely upregulated 64 miRNAs. Among them, miR‐9, miR‐21, and miR‐222 were previously reported to be involved in the regulation of *FoxO1* expression by binding the 3′UTR of *FoxO1* mRNA in cancer cells [34, 35, 36]. These three upregulated miRNAs might contribute to the β_2_‐AR stimulation‐induced decrease in *FoxO1* expression in C2C12 myotubes. Furthermore, a different set of four miRNAs (miR‐374b‐5p, miR‐7038‐3p, miR‐7016‐3p, and miR‐7a‐1‐3p) predicted to target the 3′UTR of *FoxO1*.

To investigate the functional activity of these miRNAs, we examined *FoxO1* mRNA expression in myotubes transfected with their mimics. Among the four mimics, either miR‐374b‐5p or miR‐7a‐1‐3p successfully decreased *FoxO1* mRNA expression in the C2C12 myotubes. In addition, we confirmed that the transcriptional activity of FoxO was decreased in the C2C12 myotubes transfected with these two mimics. Indeed, decreased *Atrogin‐1/MAFbx* mRNA expression was confirmed in the C2C12 myotubes transfected with either miR‐374b‐5p or miR‐7a‐1‐3p mimics. These results suggest that, in addition to the previously reported miRNAs (miR‐9, miR‐21, and miR‐222), either miR‐374b‐5p or miR‐7a‐1‐3p is rapidly upregulated by β_2_‐AR stimulation and destabilizes *FoxO1* mRNA, consequently affecting its transcriptional activity against its target gene (e.g., *Atrogin‐1/MAFbx*) in myotubes. However, TargetScan analysis also predicted that the 3′UTR of *Atrogin‐1/MAFbx* would be targeted by both miRNAs, which raises the possibility that these miRNAs directly destabilize *Atrogin‐1/MAFbx* mRNA, even though β_2_‐AR stimulation did not affect *Atrogin‐1/MAFbx* mRNA expression in myotubes within 1 h after stimulation (data not shown).

As mentioned above, norepinephrine stimulation decreases FoxO1 protein abundance via the cAMP–PKA pathway in murine skeletal muscle [25]. β_2_‐AR couples with the G protein, which activates adenylyl cyclase, catalyzing the formation of cAMP [37]. It has also been reported that β_2_‐AR stimulation by clenbuterol increased the cAMP concentration in rat skeletal muscle [38]. In this study, we confirmed that, in C2C12 myotubes, β_2_‐AR stimulation rapidly increased cAMP concentration. Then, to inhibit the β_2_‐AR agonist‐induced increase in cAMP, we employed an adenylyl cyclase inhibitor, SQ22536. Pretreatment with SQ22536 successfully canceled out the β_2_‐AR agonist‐induced increases in miR‐374b‐5p and miR‐7a‐1‐3p expression, in concert with the increase of cAMP concentration in C2C12 myotubes. In addition, pretreatment with SQ22536 also canceled out the β_2_‐AR stimulation‐induced decreases in *FoxO1* mRNA expression in C2C12 myotubes. Silveira et al. [25] reported that the *in vivo* muscle‐specific activation of PKA, a cAMP‐dependent protein kinase, inhibited FoxO transcriptional activity. These results suggest that β_2_‐AR stimulation induced either miR‐374b‐5p or miR‐7a‐1‐3p via β_2_‐AR–cAMP signaling and that these increased miRNAs in turn contributed to suppressing FoxO1 transcriptional activity in C2C12 myotubes.

It has been reported that cAMP elevation changed the expression of a large number of miRNAs. For example, in human placental cells, forskolin, an inducer of intracellular cAMP, significantly changed the expression of 116 miRNAs [39, 40, 41]. However, how β_2_‐AR–cAMP signaling increases either miR‐374b‐5p or miR‐7a‐1‐3p expression in C2C12 myotubes remains unclear. One possible explanation for this might involve the endoribonucleases (e.g., DICER and DROSHA) required for miRNA processing [42]. In isolated human endometrial stromal cells (hESCs), cAMP and medroxyprogesterone acetate treatment significantly increased DICER expression, suggesting that cAMP elevation was related to the miRNA maturation and degradation of *FoxO1* mRNA [43]. Furthermore, miR‐374b‐5p and miR‐7a‐1‐3p are involved in Ftx transcript (ENSMUSG00000086370) and heterogeneous nuclear ribonucleoprotein K (ENSMUSG00000021546) (Hnrnpk), respectively. Although the relationship between β_2_‐AR stimulation and Ftx transcript expression remains unclear, it has been reported that β_2_‐AR stimulation increased Hnrnpk protein levels in the skeletal muscle of human with high‐intensity training [44]. It was suggested that expressions of these transcripts have important implications for β_2_‐AR stimulation‐induced increase in either miR‐374b‐5p or miR‐7a‐1‐3p. Further investigations are needed to gain insight into the mechanisms by which β_2_‐AR stimulation recruits these miRNAs in myotubes.

In conclusion, β_2_‐AR stimulation by clenbuterol rapidly upregulated miR‐374b‐5p and miR‐7a‐1‐3p in myotubes and then decreased the mRNA expression of *FoxO1* via the β_2_‐AR–cAMP signaling pathway. β_2_‐AR–cAMP signaling suppresses FoxO1 transcriptional activity that would otherwise promote the expression of genes encoding *Atrogin‐1/MAFbx* mRNA, and consequently suppresses protein degradation via the dual mechanisms of altering FoxO1 protein abundance and phosphorylation in skeletal muscles.

## Conflict of interest

The authors declare no conflict of interest.

## Author contributions

SS, KN, AO, and DI conceived and designed the experiments. SS, KN, NN, and RK performed the experiments and contributed reagents/materials/analysis tools. SS, KN, and DI wrote the paper. AO and SF reviewed and revised the paper.

## Data Availability

The data that support the findings of this study are openly available in NCBI's Gene Expression Omnibus and are accessible through https://www.ncbi.nlm.nih.gov/geo/query/acc.cgi. GEO Series accession number is GSE130181.
